# Understanding drivers of human-leopard conflicts in the Indian Himalayan region: Spatio-temporal patterns of conflicts and perception of local communities towards conserving large carnivores

**DOI:** 10.1371/journal.pone.0204528

**Published:** 2018-10-05

**Authors:** Dipanjan Naha, S. Sathyakumar, G. S. Rawat

**Affiliations:** 1 Department of Endangered Species Management, Wildlife Institute of India, Dehradun, Uttarakhand, India; 2 Wildlife Institute of India, Dehradun, Uttarakhand, India; Sichuan University, CHINA

## Abstract

Human killing is the decisive and most critical expression of human-leopard conflict and needs to be addressed sensitively to maintain local support for leopard conservation in India. This research was undertaken to investigate the ecological aspects of human killing and injury, spatial characteristic and pattern of such sites, temporal and seasonal trends of attacks and perception of local communities towards leopard in the Indian Himalayan region (IHR). We surveyed two sites i) Pauri Garhwal in the western part and ii) North Bengal (Dooars) in the eastern part of IHR, compiled secondary data on human-leopard conflict records and made field visits to (N = 101) conflict sites. We also conducted (N = 186) semi-structured questionnaire surveys in each of the sites to assess perception of local communities towards leopard. We analyzed the conflict data using rare events model in a binary logistic regression framework to understand spatial patterns of such incidents for Pauri Garhwal and North Bengal. The average number of injuries and deaths to leopard attacks in Pauri was estimated to be 11 (SE 1.13) and 3 (SE 0.6) per year between 2006–2016 whereas in North Bengal it was estimated to be 70 (SE 9.2) and 1.6 (SE 0.3) respectively between 2004–2016. About 97% of the leopard attacks in North Bengal and 60% of the leopard attacks in Pauri resulted in human injuries. Majority of the leopard attack victims in Pauri were children and young people, whereas in North Bengal it was middle aged tea estate workers. Attack on humans in Pauri were recorded mostly near areas with dense scrub cover whereas in North Bengal it was reported within tea-estates. The percentage of human deaths to leopard attacks in Pauri were higher (40%) compared to a mere (3%) in North Bengal. Forty-one percent of respondents in Pauri and 75% in North Bengal were positive towards presence and conservation of leopard. A predictive risk map revealed central and northern regions of Pauri Garhwal and protected areas, peripheral areas in central and south-western dooars (North Bengal) as high “human-leopard conflict risk zones”. This analytical procedure can be adopted in other sites to identify potential human-carnivore conflict risk zones.

## Introduction

There has been an increase in severity of human-wildlife conflicts in India in the last few decades with tiger (*Panthera tigris*), Common leopard (*Panthera pardus*) and Asian elephant (*Elephas maximus*) being the three most problematic species reported to cause extensive damage to human lives, livestock and property. While an extensive protected area (PA) network and land allotted for agricultural production were cited as two major reasons [1], the real cause of escalation of conflicts in the recent years has been attributed to habitat loss, fragmentation, degradation due to increasing anthropogenic pressures, particularly development, reducing tolerance levels to wildlife, and local abundance of problem species [2]. When there are incidents of large cats such as tiger and leopard killing and injuring humans, it evokes a serious public backlash and a setback for conservation efforts. Though studies have been periodically conducted within PAs on certain aspects of ecology of such large mammals in India, extensive research on such aspects in regions where they share space with humans are limited [3–5]. Knowledge gained through such studies in human-dominated landscapes help solve complex conservation problems such as human-wildlife conflicts [6–12], where apart from the dynamics of such events, a thorough understanding of the social aspects of conflicts are essential for implementing further mitigation measures [13–19].

In India, where the interface between forests and rural inhabitations is a continuum, the leopard has adapted to live in the fringes of human habitations. Due to the behavioural plasticity, wide choice of prey and adaptability to survive on a wide range of human altered habitats, the smaller, agile and adaptable leopard is most often implicated in attacks on people [20]. Today—human-leopard conflicts are reported across India with major hotspots being Uttarakhand, West Bengal, Himachal Pradesh, Gujarat and Maharashtra [21]. Recent studies on leopard attacks on humans and livestock have been conducted [22–24] in Uttarakhand and [25,26] in North Bengal but none have compared spatio-temporal patterns and major drivers of such incidents across these sites. Gazetteers and hunting records maintained by the British officers in colonial times confirm visitation by leopard frequently near human settlements to predate on livestock and domestic animals in spite of vast wilderness areas with abundant wild prey. In the western part of this region, Uttarakhand state (Pauri Garhwal) has been historically recognised for the magnitude of human-leopard conflict, when hundreds of people were killed by leopard even in the 20^th^ century and a dozen leopards killed as man-eaters [27]. Since the last decade, there has been large scale human out-migration from the mountainous region to the plains due to lack of sustained livelihood resources. Pauri Garhwal reported an annual growth rate of -0.13 with 122 villages abandoned completely between 2001–2011 [28]. Thus present day Pauri Garhwal, is a matrix of agriculture lands, villages/towns, scrub/secondary forests and mature forests which is best suited for leopard [23]. In the eastern Himalayan foothills (dooars region of West Bengal) there has been reports of more than 700 attacks on humans by leopard between 1990–2016 [26]. The dooars region underwent rapid changes since colonial times when British planters cleared large tracts of forests for commercial tea plantations and settled tribal people from Central Indian highlands to work as daily labourers in these gardens [29].

Keeping in perspective the recurrent loss of human lives and frequent injuries by leopard attacks our objective was to assess spatial and temporal patterns of attacks and perception of local communities in two such landscapes across the Indian Himalayan Region (IHR). We evaluated the (1) present and past trends in intensity of conflict (2) documented spatial distribution of human-killing and injury related incidents and identified ecological variables, potential hotspots of conflict, (3) identified human activities that make people vulnerable to attack, (4) socio-economic condition of local people, and (5) perception of local people towards leopard. For the present study, we did not investigate drivers of livestock depredation by leopard. We used the results to develop recommendations for management interventions in mitigating human-leopard conflict in the IHR.

## Materials & methods

### Study area

**Fig 1:**
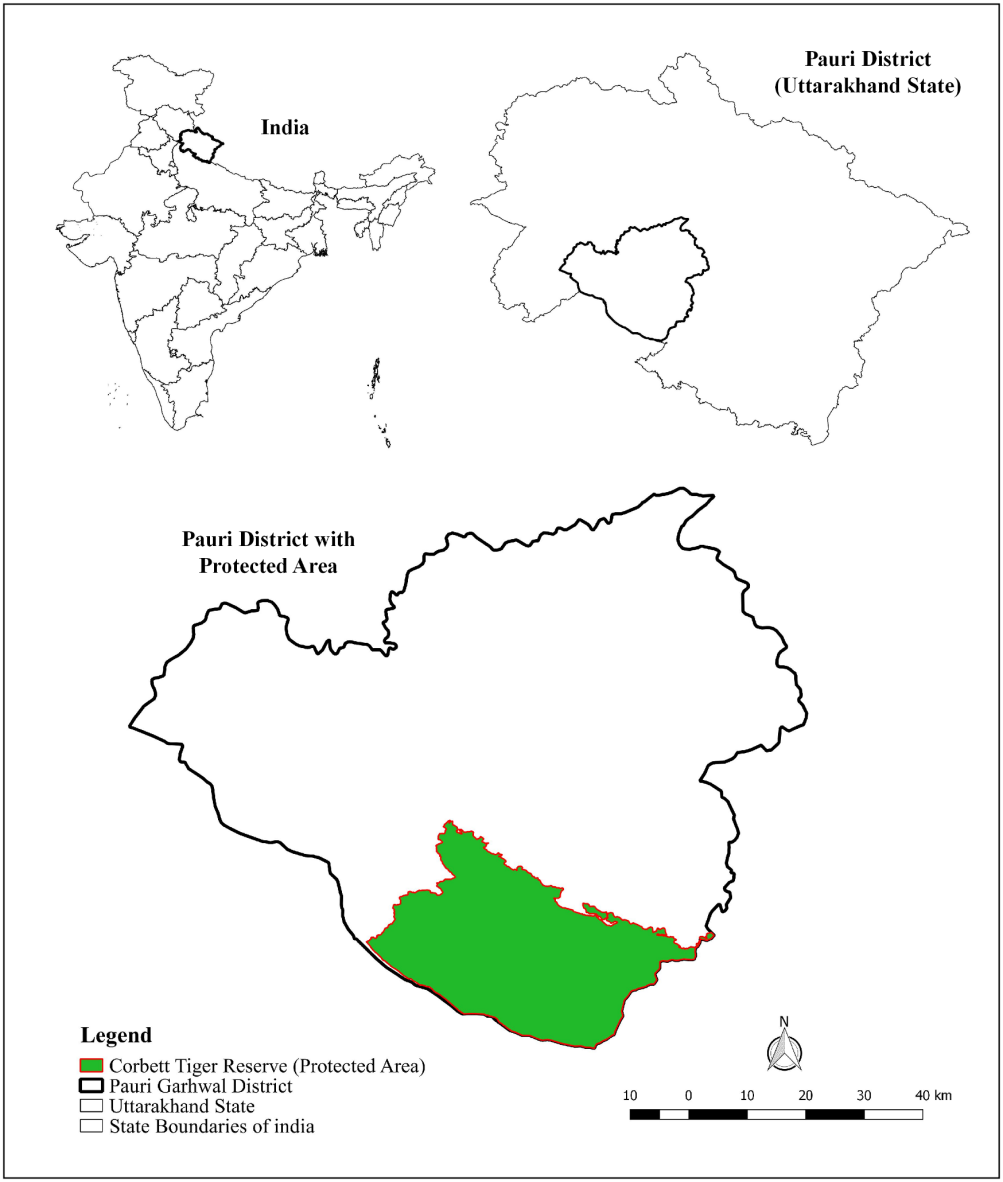
Map depicting location of study area (Pauri Garhwal) within Uttarakhand State, India.

**Fig 2:**
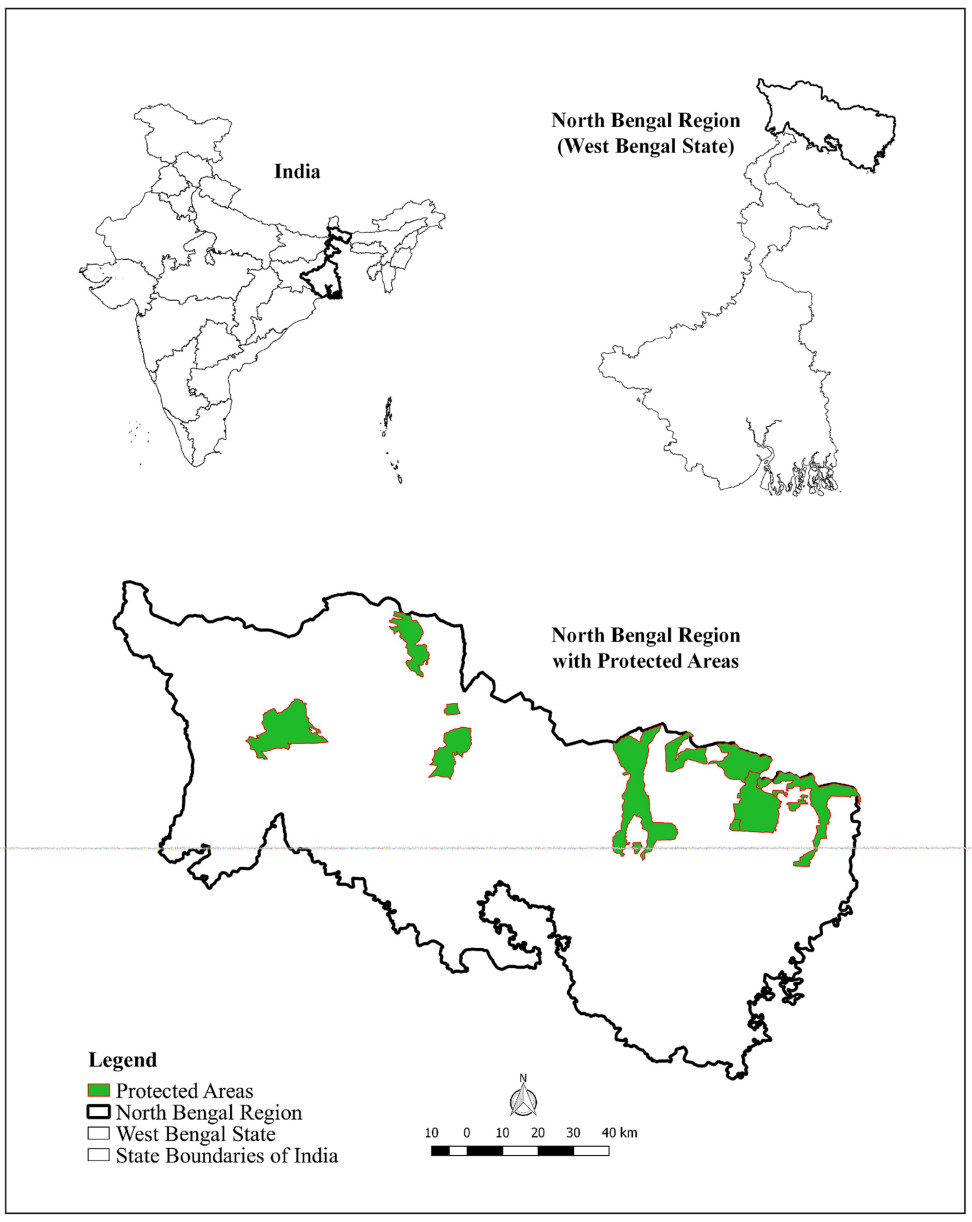
Map depicting location of study area (North Bengal) within West Bengal State, India.

The two study sites are spread across the western and eastern IHR Figs 1 & 2. These two sites in the IHR differ in certain standard parameters such as (i) human population, (ii) variation in altitude, (iii) forest cover, (iv) precipitation, (v) major land use patterns, and (vi) livestock population.

Pauri Garhwal is a district in Uttarakhand state and within close proximity to PAs such as Corbett and Rajaji Tiger Reserves. This district falls within Biogeographic Zone 2 (Himalaya) and Biotic Province 2B (Western Himalaya) as per the Biogeographic Classification of India [30]. This district is part of the lesser and middle Himalaya with an altitudinal range between (200–3200 m). It has a forest cover of 64% with the area under moderate dense forest being almost 2000 km^2^, followed by scrublands and open forests [31]. Average rainfall in the district is 218 cm. with majority received during monsoon.

The entire district is rugged and mountainous. The human population density is low, 110 persons per km^2^ (Census 2011, data accessed on June 2018) with the local inhabitants of Pauri district predominantly agrarian with other major occupations being horticulture, livestock farming and cottage industries [23]. The livestock density of this region is 58 per km^2^ (Livestock Census, 2012). Major mammalian fauna of Pauri region are Asiatic black bear (*Ursus thibetanus*), common leopard (*Panthera pardus fusca*), barking deer (*Muntiacus muntjak*), goral (*Nemorhaedus goral*), sambar (*Cervus unicolor*), wild pig (*Sus scrofa*), rhesus macaque (*Macaca mulata*) and common langur (*Presbytis entellus*).

The dooars (North Bengal) region is a part of Jalpaiguri District of West Bengal within close proximity of PAs such as Chapramari Wildlife Sanctuary and Gorumara and Neora Valley National Parks. The dooars fall within Biogeographic zone 2 and Biotic provinces 2C and 7B [30]. The predominant forest types are Northern Tropical Semi-Evergreen and Tropical Moist Deciduous forests [32]. The North Bengal landscape underwent rapid change in the 1850’s when British cleared large tracts of forests for commercial tea plantations, brought tribal people from Central India and engaged them as daily labors to work in tea gardens. Subsequently after Independence and creation of Bangladesh, Government of India settled large number of refugees within this region [33].

The forest areas were cleared to make way for human settlements and thus today the landscape is a matrix of tea gardens, villages, urban settlements interspersed with small PAs [29]. There are 70 tea estates in this region with the recorded forest area in recent times being 46% with an area of 1700 km^2^ under open forests and scrublands [19]. The average human population density is 701 persons per km^2^ (Census 2011, http://jalpaiguri.gov.in/html/census. html, accessed on June 2018). The region receives an average annual rainfall of 3160 mm with an average altitude of 200 m. The major mammalian fauna of this region are the endangered one-horned rhinoceros (*Rhinoceros unicornis*), Asian elephant, gaur (*Bos gaurus*), sambar (*Rusa unicolor*), chital (*Axis axis*), rhesus macaque (*Macaca mullata*) and a host of diverse fauna and flora with leopard being the apex predator and only large carnivore present [25].

### Field methods

#### Compiling conflict data and field visits

In order to understand the nature and extent of human-leopard conflicts we compiled Forest Department wildlife damage compensation records, published literature, newspaper reports available regarding the number of human death and injuries, leopard deaths in Pauri Garhwal and North Bengal for the past 16 years (2000–2016). Based on these records we visited (N = 43) sites within Pauri Garhwal and (N = 58) in North Bengal where leopard have attacked humans recently between June 2016 to October 2017. Subsequently we mapped the gps locations in Arc GIS 10.2. We also inquired about the details of such incidents from family members of leopard victims, companions, local people and forest personnel who were present or had visited these sites after the attacks happened. We also noted other details such as age and occupation of victim, time and month of attack, activity during attack, vegetation type and altitude of conflict site as well as whether the attack resulted in death or injury. We conducted perception based surveys of local communities to understand social drivers of conflicts within both Pauri and North Bengal. A total of (N = 182) respondents in Pauri and (N = 186) in North Bengal were interviewed between January 2017 and April 2018 using closed and open ended semi-structured and structured questionnaires [34,35] to assess their socio-economic condition, dependence on forest resources and attitudes towards leopard. The initial questions were related to simple demographic information to ease respondents into the interview session. Questions were repeated several times if the respondents had problem in comprehending and a response was noted down only when there was no ambiguity.

A family was treated as the basic unit for the purpose of this study, with only one respondent from a family being interviewed. The structured questionnaire used was divided into three main sections viz., (i) the first section primarily dealt with the demographic details (age, gender, caste, education level, household structure etc.) and visual assessment of the economic condition of the interviewee. (ii) The second part dealt with socio-economic questions pertaining to land holding, occupation and livestock owned and (iii) the final section comprised of questions regarding knowledge of leopard, human-leopard interactions, perception towards leopard conservation and suggestions for mitigating conflict. Responses from the questionnaire survey were analyzed to evaluate basic statistics regarding socio-economic well-being, primary source of livelihood and perception of people towards leopard (S1 Appendix).

#### Predation risk mapping

The attack data from the compiled records were analyzed to arrive at spatial-temporal pattern of such attacks. We used chi-square analysis (α = 0.05) [36] to compare attack events between seasons, months of the year, time of the day and different age class and occupation of leopard attack victims. Due to logistic constraints, we collected conflict data only from central and northern eastern parts of Pauri district, and central and eastern parts of North Bengal. The two study sites were stratified into 2 km^2^ grids using Arc GIS 10.2. Conflict information was collected on a presence/absence binary coding basis with a grid having a conflict incident recorded as ‘1’ and ‘0’ if absent. A total of 14 predictor variables (independent variables) were selected based on their ecological importance Table 1. Area of major vegetation types (Dense forest, Open Forest, Moderately Dense Forest, Scrubland) were derived from the 23.5 m spatial resolution forest cover map [37]. Length of roads and drainage within each grid were extracted using the Roads and Drainage layers obtained from Digital Chart of the World [38]. Altitude of each grid centroid was generated from the 90 m spatial resolution digital elevation maps [39]. Annual mean precipitation and temperature data for Pauri and North Bengal were extracted from World BIOCLIM data [40]. Human footprint data was generated using the human census data 2011 (Census Data 2011.) available at the resolution of a village. Night light data for the two sites were derived using 1000 m spatial resolution night-time visible lights data of India [41]. After extraction, we standardized all these data using z transformations. As data showed over dispersion due to excessive number of zeroes or absence locations, we fitted the rare events logistic regression using the function relogit [42] in package “zelig” in (R 3.4.0). The rare events regression procedure estimates the model as standard logistic regression with the output estimates corrected for the bias that occurs when the sample is small or the observed events rare. We selected the final model based on the lowest AIC value [43]. The logit link in a logistic regression is used to model the log-odds of an event occurring. Here we used a simple logistic regression with a dichotomous confounder (Z) and a dichotomous exposure E [44]. This can also be used for multiple categorical or continuous variables and thus following a logistic regression model by maximum likelihood we computed probability of conflict $\hat{\text{p}}\text{ez}$ for any E = *e* and Z = *z* as follows:

$$\hat{P}ez=\text{exp}\left[\hat{\alpha }+{\hat{\beta }}_{1}\times e+{\hat{\beta }}_{2}\times z\right]/\left(1+\text{exp}\left[\hat{\alpha }+{\hat{\beta }}_{1}\times e+{\hat{\beta }}_{2}\times z\right]\right)$$

where $\hat{\alpha }$, ${\hat{\beta }}_{1}$ and ${\hat{\beta }}_{2}$ are the estimated regression coefficients.

**Table 1:** Independent variables considered for predictive risk modelling and mapping using rare events model.

Serial No.	Independent Variable
1	Length of road
2	No of Road
3	Length of Drainage
4	Area under water
5	Human footprint
6	Night light
7	Altitude
8	Annual Mean Temperature
9	Very Dense Forest
10	Open Forest
11	Moderately Dense Forest
12	Scrubland
13	Non-Forest
14	Annual Mean Precipitation

Using this probability values conflict risk maps were prepared in Arc GIS 10.2.

## Results

### Human-leopard conflict

Based on the forest department records, in Pauri Garhwal, a total of 159 attacks on humans were registered between 2006 and 2016. The mean number of humans killed by leopard per year in Pauri Garhwal during this period was estimated to be 3.5 (SE 0.91). Apart from the deaths, an average of 11 persons were injured by leopard attacks during the same period. In North Bengal, a total of 121 attacks on humans were registered between 1993–1997, 243 between 2001–2008 and 420 between 2011–2016. The average number of injuries between 2011–2016 were estimated to be 70 (SE 9.4) whereas deaths were 1.6 per year (SE 0.3) between 2004–2016. A total of 121 leopards were killed in Pauri Garhwal either in retaliation by local communities or declared as man-eaters and shot with a range of 2–16 per year between 1990–2005. Leopard killed in retaliation or shot dead by forest officials in North Bengal during this period was negligible and were thus not reported.

### Seasonal and temporal variation of leopard attacks

Though there was no significant difference in seasonal pattern of leopard attacks on humans in Pauri Garhwal (*χ*^2^ = 2.68, df = 3, p-value > 0.05), 30% attacks occurred between February to April and 27% between May to July. Based on our field surveys (N = 43), 28% and 30% of the incidents in Pauri were recorded between 0900h and 1200h and 1500h to 1800h respectively (*χ*^*2*^ = 76.94, df = 7, p-value < 0.05). In North Bengal, there was significant seasonal variation in pattern of leopard attacks (*χ*^*2*^ = 37.38, df = 3, p-value < 0.05) with 24% and 49% attacks recorded between November to January and February to April respectively. Based on our field surveys, 37% and 39% of leopard attacks were documented between 0900h to 1200h and 1200h to 1500h respectively (*χ*^*2*^ = 151.29, df = 7, p-value < 0.05). Fifty seven percent of the attacks on humans in Pauri Garhwal occurred within an altitudinal range of 1000–1500 m whereas in North Bengal all attacks were recorded between 100–500 m (S1 Fig).

### Age, profession and activity of leopard attack victims

Twenty-three percent of the leopard attack victims (both injury and deaths) in Pauri Garhwal were between 11–20 years in age followed by 21% between 1–10 years and 18% in the middle age group of 31–40 years. Majority of the victims who died due to leopard attacks were children and young people, 41% in the age class of (1–10) years, followed by 24% in the age category of 11–20 years. During field visits and informal interactions, family members, friends responded that the victims were either walking back from school, market, collecting firewood or working alone in agriculture lands. Fifty-two percent of these victims were males and rest females. When attacked most of the victims were solitary or in groups comprising of <3 people. Majority (76%) of the leopard attacks sites in Pauri Garhwal had medium to dense shrub cover.

In North Bengal, an overwhelming 90% attacks on humans occurred in tea estates. Seventy-eight percent of the victims who sustained injuries were tea estate workers by profession. Fifty-eight percent of these victims were males and rest females. Majority of the victims were working in small scattered groups of < 2 persons when leopard attacked them in tea estates. The attacks happened when workers were busy plucking tea leaves, spraying insecticides or watering the tea plants.

### Predictor variables

Out of the 14 predictor variables, precipitation, temperature and altitude were ecologically significant variables in Pauri Garhwal whereas in North Bengal open forests, scrublands significantly affected leopard attacks on humans. The best model was selected based on the lowest AIC values Tables 2 and 3. In Pauri Garhwal, precipitation, dense forests and altitude of 1500 m and beyond were significant ecological variables responsible for leopard attacks Table 4 whereas in North Bengal it was open forests, length of water bodies, streams and altitude < 1000 m Table 5. Human-leopard conflict probability was inversely proportional to altitude with such incidents declining with increasing elevation in North Bengal. Some of the site-specific characteristics are provided as Table 6.

**Table 2:** Comparison of predictive models showing probability of human-leopard conflict risk, (Pauri Garhwal).

Model	AIC	ΔAIC	K
Ψ(N.light+H.F+P+Alt+AT+L.R+N.R+D.F+M.DF+O.F+S.Land+N.F+L.D)	330.08		12
Ψ(H.F+P+Alt+AT+L.R+D.F+M.DF+O.F+S.Land+L.D)	334.06	3.98	10
Ψ(N.light+H.F+P+AT+Alt+L.R+N.R+L.D+W+D.F+O.F+S.Land+N.F+MDF)	338.89	8.81	14

Ψ: Probability of human-leopard conflict; N.light: Night Light; H.F: Human Footprint; P: Annual Mean Precipitation; Alt: Altitude of Grid Centroid; L.R: Length of road; A.T: Annual Mean Temperature; L.R: Length of Road; N.R: Number of roads; D.F: Area under Dense Forest; M.DF: Area under Moderate Dense Forest; O.F: Area under Open Forest; S.Land: Area under Scrubland; N.F: Area under Non-Forest; W: Area under water; L.D: Length of drainage; K: No of parameters.

**Table 3:** Comparison of predictive models showing human-leopard conflict risk probability (North Bengal).

Model	AIC	ΔAIC	K
Ψ(N.light+H.F+P+Alt+L.R+D.F+O.F+S.Land+N.F+W+L.D)	308.93		11
Ψ(N.light+H.F+P+Alt+L.R+D.F+O.F+S.Land+N.F+L.D)	309.19	0.26	10
Ψ(N.light+H.F+P+Alt+AT+L.R+N.R+D.F+M.DF+O.F+S.Land+N.F+W+L.D)	310.15	1.22	14

Ψ: Probability of human-leopard conflict; N.light: Night Light; H.F: Human Footprint; P: Annual Mean Precipitation; Alt: Altitude of Grid Centroid; L.R: Length of road; A.T: Annual Mean Temperature; L.R: Length of Road; N.R: Number of roads; D.F: Area under Dense Forest; M.DF: Area under Moderate Dense Forest; O.F: Area under Open Forest; S.Land: Area under Scrubland; N.F: Area under Non-Forest; W: Area under water; L.D: Length of drainage; K: No of parameters.

**Table 4:** Beta-coefficient values of predictor variables of best model, Pauri.

Coefficient	Estimate	Std. Error	Z Value	Sig (P < 0.05)
Intercept	-5.43	0.46	-11.84	<2e-16
Length Road	0.15	0.16	0.91	0.36
Length Drainage	-0.183	0.22	-0.85	0.39
Human footprint	0.08	0.21	0.40	0.69
Night light	0.29	0.38	0.77	0.44
Altitude	1.14	0.30	3.75	0.00
Temperature	0.044	0.38	0.12	0.91
Precipitation	-0.83	0.22	-3.84	0.00
Dense Forest	1.38	0.83	1.66	0.09
Scrubland	-0.037	0.27	-0.14	0.89
Open Forest	0.19	0.41	0.47	0.64
Non -Forest	0.62	0.79	0.78	0.43
Moderate Dense Forest	0.33	0.59	0.55	0.58

**Table 5:** Beta coefficient values of predictor variables of best model, North Bengal.

Coefficients	Estimate	Std. Error	Z value	Sig (P < 0.05)
Intercept	-4.76	1.34	-3.54	0.00
Night light	0.01	0.23	0.05	0.96
Human footprint	0.28	0.22	1.29	0.19
Precipitation	-0.06	0.65	-0.09	0.92
Altitude	-1.26	1.42	-0.89	0.87
Length Road	-0.22	0.18	-1.24	0.22
Dense Forest	1.16	1.96	0.59	0.55
Open Forest	1.82	0.94	1.93	0.05
Scrubland	0.30	0.19	1.55	0.12
Non-Forest	0.96	1.11	0.86	0.398
Water	-0.10	2.05	-0.05	0.96
Length of Drainage	0.34	0.21	1.65	0.09

**Table 6:** Comparison of parameters within the two sites Pauri Garhwal and North Bengal.

Number	Variable	Pauri Garhwal	North Bengal
**1**.	Human Density per km^2^	110	700
**2**.	Livestock Density per km^2^	58	340
**3**.	Altitudinal Range (m)	200–3200	50–2400
**4**.	Major Forest Type^1^	Moderate dense forest	Scrubland and open forest
**5**.	Forest Cover Percent^1^	64	46
**6**.	Wild Prey Density per km^2^	NA	56*
**7**.	Common Leopard Density	NA	NA
**8**.	Major wild prey	^#^Barking deer, wild pig	*Barking deer, wild pig, rhesus macaque
**9**.	Major Occupation of people	^#^Government Service, Agriculture	*Tea Estate worker, livestock farming, agriculture

^1^ Data are from Forest Survey of India Report, 2017.

* Kshettry et al. 2018.

^#^ Goyal et al. 2007.

### Human-leopard conflict risk zones

**Fig 3:**
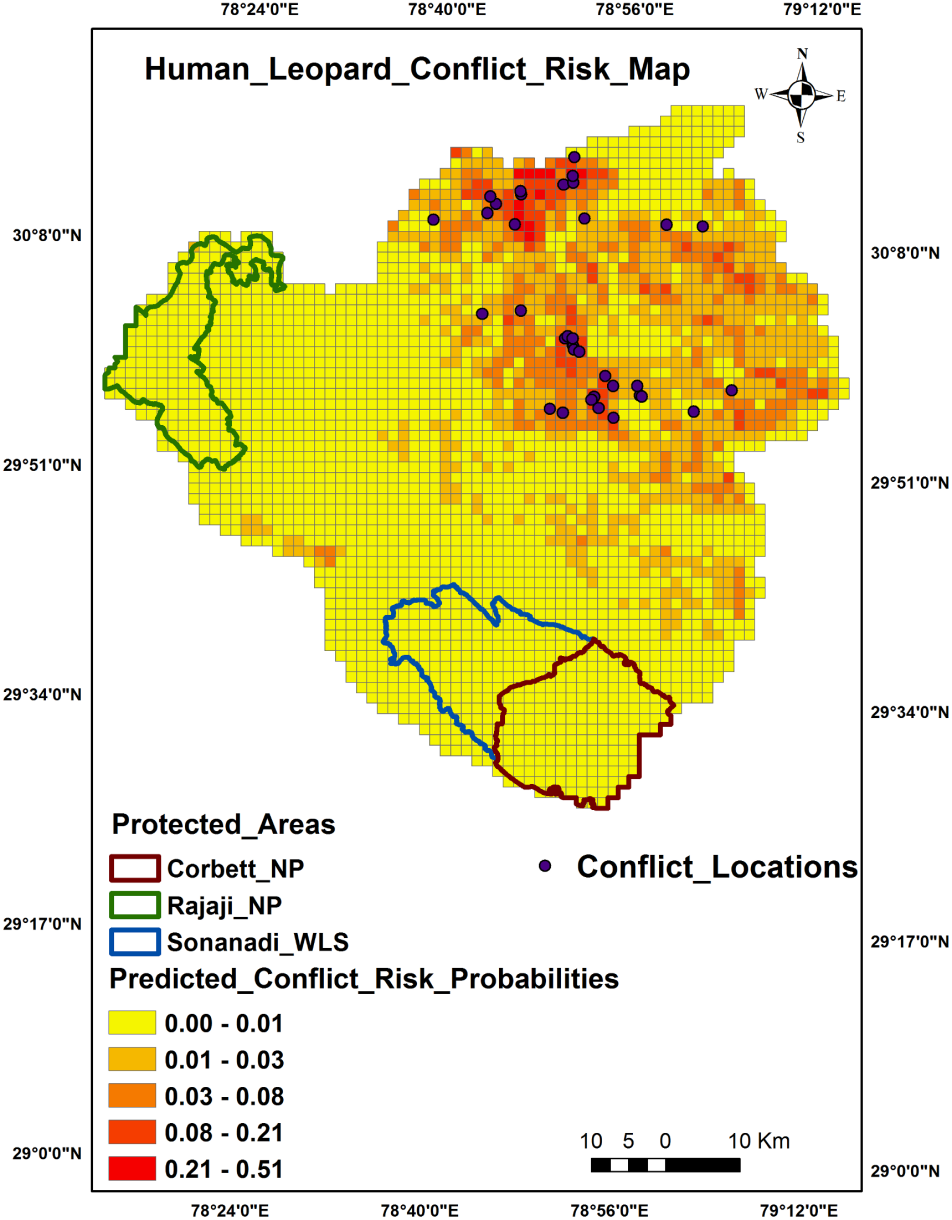
Human-leopard conflict predictive risk map, Pauri Garhwal.

**Fig 4:**
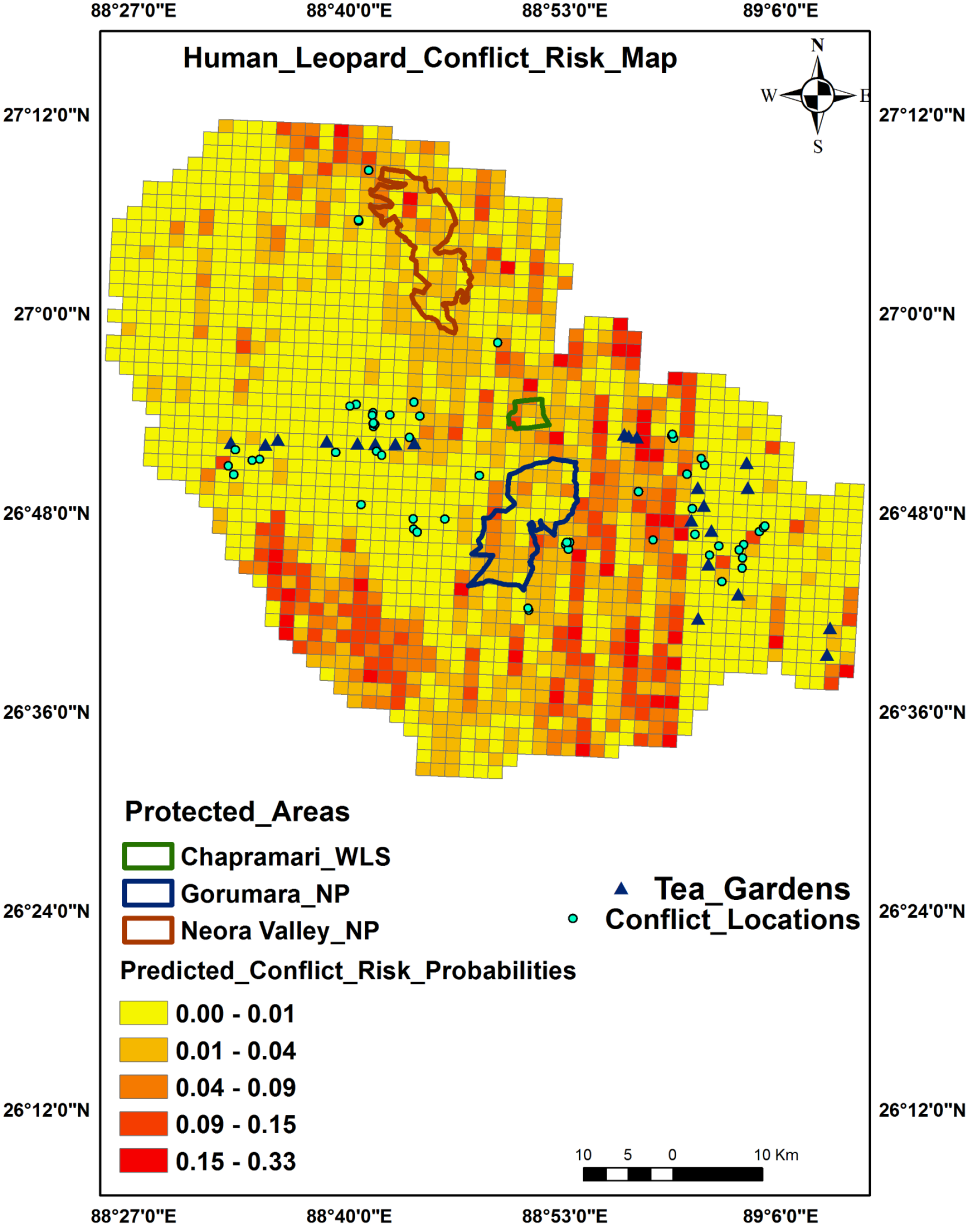
Human-leopard conflict predictive risk map, North Bengal.

Predictive maps showed central and northern regions of Pauri Garhwal as conflict risk hot spots (Fig 3) whereas for North Bengal it was the central and south western zones adjacent to PAs (Fig 4). The sites around PAs in North Bengal showed higher probabilities of conflicts and could be due to the presence of secondary forests and tea estates. A comparison of major variables between the two Himalayan sites are provided as Table 6.

### Socio-economic condition of respondents

In Pauri Garhwal majority of the respondents were middle aged (48 years SE 1.09) whereas for North Bengal they were slightly younger (38 years SE 1.08). Among the respondents interviewed in Pauri 20% were illiterate, 41% had formal education and 15% were graduates. On the contrary, in North Bengal, 43% were illiterate followed by 33% who didn’t complete secondary education, 22% who completed secondary education and only 2% having completed graduation. Some of the other parameters recorded during the interviews are provided as pie charts (S2 Fig).

Approximately 58% percent of respondents were agriculturalists in Pauri whereas in North Bengal they were 46% tea estate workers by profession (S3 Fig). The average annual income of agriculturalists was estimated to be INR 129,024, (= US $ 1886) whereas that of tea estate workers ranged between INR 36,000–48,000 (= US $526–700). Average livestock possessed per household was 13 (SE 8.23) in Pauri and 3 in North Bengal (SE 0.3). Average landholding in Pauri Garhwal was 977 (SE 706.2) m^2^ whereas in North Bengal it was 2127 m^2^ (SE 281.2). Based on the questionnaire surveys, average annual livestock depredation per household by leopard was estimated to be 3 in Pauri and 1 in North Bengal. The primary reason cited by respondents for decline in agriculture production in Pauri region was a rise in crop depredation by wild pigs and rhesus macaques (62%) whereas rest 15% felt it was shortage of irrigation water.

### Attitude of respondents towards leopard and human-leopard conflict

An equal proportion of people 41% were positive and negative towards leopard in Pauri Garhwal whereas in north Bengal a majority 75% of the respondents were positive towards leopard (S3 Fig). Occupation wise 65% of tea estate workers in North Bengal and 70% agriculturists in Pauri disliked leopard.

Majority of respondents 89% felt that leopard attacks on humans in Pauri were intentional without being provoked, whereas 76% of the people in North Bengal opined that leopard attacks were accidental and in self-defense. Forty-two percent of the people interviewed in Pauri stated that the role of leopard was to predate and reduce wild and domestic prey followed by 40% who stated that had no role in the wild. Almost 99% of the people in Pauri orated that conversion of agriculture lands into secondary forests, scrublands have provided refuge to leopard near human settlements and thus conflicts have been reported near villages in recent times. Forty-eight percent of the people in North Bengal had no knowhow of the role of leopard, 24% stated that they helped maintain ecological balance whereas 17% believed their primary role was to destroy and subdue other animals. Fifty percent of the respondents in Pauri and 52% of the respondents in North Bengal stated that decline of wild prey was primary reason for leopard attack on humans followed by 24% and 27% who believed that easy availability of domestic prey i.e. livestock attracted leopard towards human habitation (S4 Fig).

Forty-three percent of respondents in Pauri stated that there was no solution to mitigate leopard attacks on humans followed by 18% who stated that increasing compensation amount would help. In North Bengal, 60% of the respondents stated increasing vigilance while working and adapting early warning systems in tea estates as likely measures to reduce conflicts (S4 Fig). To reduce livestock depredation respondents opined to use predator proof enclosures and lights around households in Pauri whereas in North Bengal people vouched for relocating problem animals, introducing native wild prey in forests and installing predator proof enclosures.

## Discussion

The present study explores the nature of human-leopard conflicts and perception of local communities across the western and eastern Indian Himalayan region. The predictive map highlights potential human-leopard conflict zones and helps formulate mitigation measures for these sites. The number of injuries to leopard attacks were much higher in North Bengal compared to Pauri Garhwal, whereas deaths to such attacks were higher in Pauri. The total number of deaths and injuries to leopard attacks in Pauri district have reduced to 154 between 2004 and 2016 compared to 556 reported between 1998–2005 [23]. Compared to the 1990’s when there were only 121 leopard attacks in 4 years in North Bengal it has increased substantially to 805 in the last 13 years between 2004–2016. In North Bengal the primary reason of rise in leopard attacks could be the large scale expansion of tea estates and subsequent increase in human activities within the gardens [26].

It is a matter of scientific curiosity as to why conflicts suddenly reduced in Pauri Garhwal but there could be several reasons contributing to this significant decline. One of them could be the large-scale killing of leopard especially females between 1990–2005 [23] by forest officials and retaliatory killing by local communities. Though remedial harvesting hypothesis suggests that removal of large cats at small scale from certain sites does not necessarily suppress their densities or reduce the population and other individuals occupy the vacant territories left by the residents [45] but in Pauri female leopards being removed in majority [23], the annual growth rate of the population might have been affected. Hunting of adult females have been reported to have important implications for the long term viability of leopard populations compared to adult males [46] due to a male biased dispersal [47]. Though studies on brown bears and wolves in Europe have documented increased reproduction in populations with long persecution histories as compensation to loss in retaliatory killings [48] but it has not been reported in felids. Moreover, carnivore populations subjected to lethal control could have other ramifications such as i) relatively young age structure ii) lack of hunting skills in sub-adults, cubs due to removal of adult females and thus iii) increased dependence on easy prey i.e. livestock for food [49,50]. It will be interesting to investigate population dynamics and litter size of the leopard population in Pauri Garhwal. The other primary reason of decline in conflicts could be the large scale out-migration of people from the hills [28] abandoning agriculture lands and households. Studies conducted in Maharashtra have cited human intervention of capture and release of leopards to far-off sites disrupting their socio-biology as principal reason of increased attacks on people. The study also concluded that abundance of leopard and humans were not the primary drivers of conflicts [20].

The local communities believed that leopard attacks in Pauri were predatory in nature whereas in North Bengal it was mostly accidental. During the severe epidemic influenza in 1918, people of Garhwal mostly hindus disposed of human corpses in the forests and leopard being scavengers opportunistically fed on these dead bodies [27]. Over a period of time they developed a taste of human flesh and after the epidemic subsided due to lack of such corpses in the forests, leopard started killing humans frequently in the hills. This rise in attacks on humans in Pauri Garhwal was hypothesized to be a learned behavior among the leopard population which was passed on from parents to cubs and thus man-eating persisted [23]. In North Bengal, incidents of man-eating has been very rare and large scale game hunting of leopard was quite frequent till the 18^th^-19^th^ century by British planters and the Cooch Behar royal family [51]. Such historical events of game hunting in this region may have led leopards to relate people with fear of persecution. Majority of the attacks on humans in Pauri were diurnal with leopard attack victims mostly children who were going home unaccompanied either after school or returning form market alone. This was similar to findings of previous studies by [22,23] in Pauri and [52] in Maharashtra. It has been observed that households in Pauri Garhwal are located far away from major roads/markets and the trails, paths are usually covered with dense scrub and weed providing ideal cover for ambush predators like leopard. Previous studies had documented a high proportion of attacks on women during dawn and dusk primarily due to lack of toilets in the households [22] but we had very few 2% of cases, when people were outdoors for defecation purpose and were attacked by leopards. A similar study [53] in the western Himalayan state of Himachal Pradesh documented majority of the leopard attacks on unsupervised children after dusk. A high proportion of these incidents were recorded in the vicinity of villages and crop fields. In the mid-mountainous region of Nepal majority of leopard attacks on humans were recorded outside PAs and in the vicinity of human settlements [54]. In North Bengal too, attacks happened between 0800h–1600h in the winter months within tea estates which are similar to findings of a previous study [26]. The tea estate workers were mostly in scattered groups of 2–3 members during such incidents. Seventy-eight percent of the victims were workers busy in spraying pesticides, plucking new flush of leaves or watering the plants in tea gardens. Tea gardens provide ideal day refuge and cover for littering and raising leopard cubs and thus attacks on humans within these sites are accidental and in self-defense. Characteristic of carnivore attacks are site specific with diurnal nature of tiger attacks on fishermen and honey collectors recorded in the Sundarban [55] whereas in Africa and western India lion attacks occur at night when people are busy guarding cattle [56, 57]. In the Terai floodplain of Nepal, most human deaths by tiger attacks occurred within a proximity of one kilometer of the forest edge because of the high intensity of human use as compared to the forest interior [58], whereas tiger attacks were mostly reported from suboptimal habitats near forest edges of Sumatra [59]. Some of the most significant reasons cited for attacks on humans were diurnal nature of tiger activity and preferential use of certain habitat types in Sundarban mangroves recorded through satellite telemetry [55] and radio-collaring of leopard within both these sites should also be undertaken to understand their movement and resource utilization patterns.

Majority of the respondents within both study sites cited decline in wild prey as the primary driver of human-leopard conflicts and we are planning to undertake assessment of prey base soon. Diet selection studies conducted by [23, 60] have documented leopard to be surviving majorly on livestock in Pauri and North Bengal. Studies conducted in Maharashtra [61] highlight dogs as an essential component of leopard diet in human-dominated landscapes, whereas [62] reported them to be preying on species within a range of 10–40 kg with even rodents as part of their diet [63–65]. North Bengal region being situated at the foothills of the bio-diversified Eastern Himalaya comprises of tall grasslands and sal dominated forest areas, thus ideally supporting more abundant and diverse wild prey compared to the rugged mountainous region of Pauri Garhwal. A study conducted in 2016–2017 estimated dog density to be 0.8 per km^2^ (SE 0.4) in this North Bengal region [66]. The average livestock density of Pauri is 58 per km^2^ [22] which is much lower than the 340 per km^2^ reported for North Bengal region [60]. Due to the large-scale outmigration of people from the hills few men capable of livestock herding have been observed in the villages. Now due to lack of adequate manpower there is considerable number of unattended cattle near villages which could potentially attract leopards near human habitations and act as a prey base in Pauri Garhwal. These frequent visitations near human settlements could also have triggered attacks on humans especially children. Leopard attacks on humans in Nepal are attributed to be a combination of decline in wild prey, scarcity of water and lack of supervised livestock herding and guarding practices in villages [54].

Though it is believed that education broadens people’s perspective [67] and poverty, low literacy, meagre income shape negative perception towards carnivores as with tiger in Sundarban [68] our results suggest otherwise. This is largely due to the strong foundation of Joint Forest Management (JFM) activities [69] that have long been practiced in North Bengal leading to better awareness and participation of local communities in conservation and management of biodiversity.

The reasons for negative perception of leopard in Pauri could be a combination of (i) conflict since historic times, (ii) emotional stigma of losing a family member/friend/neighbor, (iii) recurring financial loss due to livestock depredation, frequent crop damage done by wildlife, (iv) lack of sustainable livelihood opportunities and outmigration, and (v) limited mitigation measures to reduce such incidents. On the contrary there were (i) minimal human deaths to leopard attacks and no historical human-leopard conflict incidents in North Bengal, (ii) cultural and religious attachment towards wildlife, (iii) increased awareness about leopard, its behavior and positive attitude towards the species and (iv) trust in mitigation measures adopted by local forest and wildlife officials. These seems to be the primary social drivers of conflicts as has been documented with large felids worldwide [70,71]. Our qualitative data gathered through the questionnaire surveys are indicative of these primary social drivers listed above and further research on understanding these drivers should be undertaken in the near future.

This study demonstrates that predation risk models can offer valuable insight into the drivers and spatial patterns of human-wildlife conflict across a landscape [72, 73]. As depicted by the probability risk map, certain sites near the northeastern and central part of Pauri district and central, south western part of North Bengal region showed increased chances of leopard predation risk on humans. Since resources available to mitigate human-carnivore conflicts are limited, it is of immense importance to identify hotspots where humans are injured or killed. These hotspots should be intensively monitored by forest and wildlife officials with concentration of mitigation measures to reduce loss of human lives and retaliatory killing of predators [74, 75].

Based on the findings of this present study, we conclude that patterns of carnivore attacks on humans are site specific and there might be numerous socio-ecological drivers of such incidents. We recommend that community-based carnivore conflict mitigation measures be implemented immediately in Pauri region in collaboration with local administration and village communities. Outreach programs focusing on biology of leopard, patterns of leopard attacks, awareness about native fauna should be conducted regularly targeting the community heads, children and women of this region. Non-lethal approaches such as use of wildlife deterrents and formation of Village Response Teams in conflict hotspots has to be encouraged to reduce retaliatory killings of leopard in Pauri Garhwal. The complex issue of large-scale human outmigration from the mountains has to addressed by the local administration and sustainable livelihood opportunities has to be provided to the local communities. In North Bengal due to accidental nature of attacks, early warning mechanism such as setting up of motion-enabled sensors with sirens/alarms in tea gardens can be initiated by forest department and the local administration. Thus the fundamental challenge to conserve leopards in the human-dominated Himalayan region will be developing resilience and tolerance of local communities through outreach programs, trust building measures ultimately reducing the need for retaliatory predator control measures.

## Supporting information

S1 Fig Pie Charts depicting site specific characteristics of leopard attacks on humans in the Indian Himalayan Region. (TIFF)

S2 Fig Comparison of socio-economic condition of respondents across the two study sites. (TIFF)

S3 Fig Comparison of socio-economic condition of respondents across the two study sites. (TIFF)

S4 Fig Comparison of attitude of respondents across the two study sites. (TIFF)

S1 Appendix Questionnaire sheet. This datasheet was used to record socio-economic and perception data of local communities in Pauri Garhwal and North Bengal.
(DOCX)

S1 Dataset Data of covariates used for regression analysis with presence/absence information. (XLSX)
