# Supplementary figures and images for: Understanding drivers of human-leopard conflicts in the Indian Himalayan region: Spatio-temporal patterns of conflicts and perception of local communities towards conserving large carnivores
Source: PLoS One. 2018 Oct 5;13(10):e0204528. doi: 10.1371/journal.pone.0204528 (PMC6173383; doi:10.1371/journal.pone.0204528)

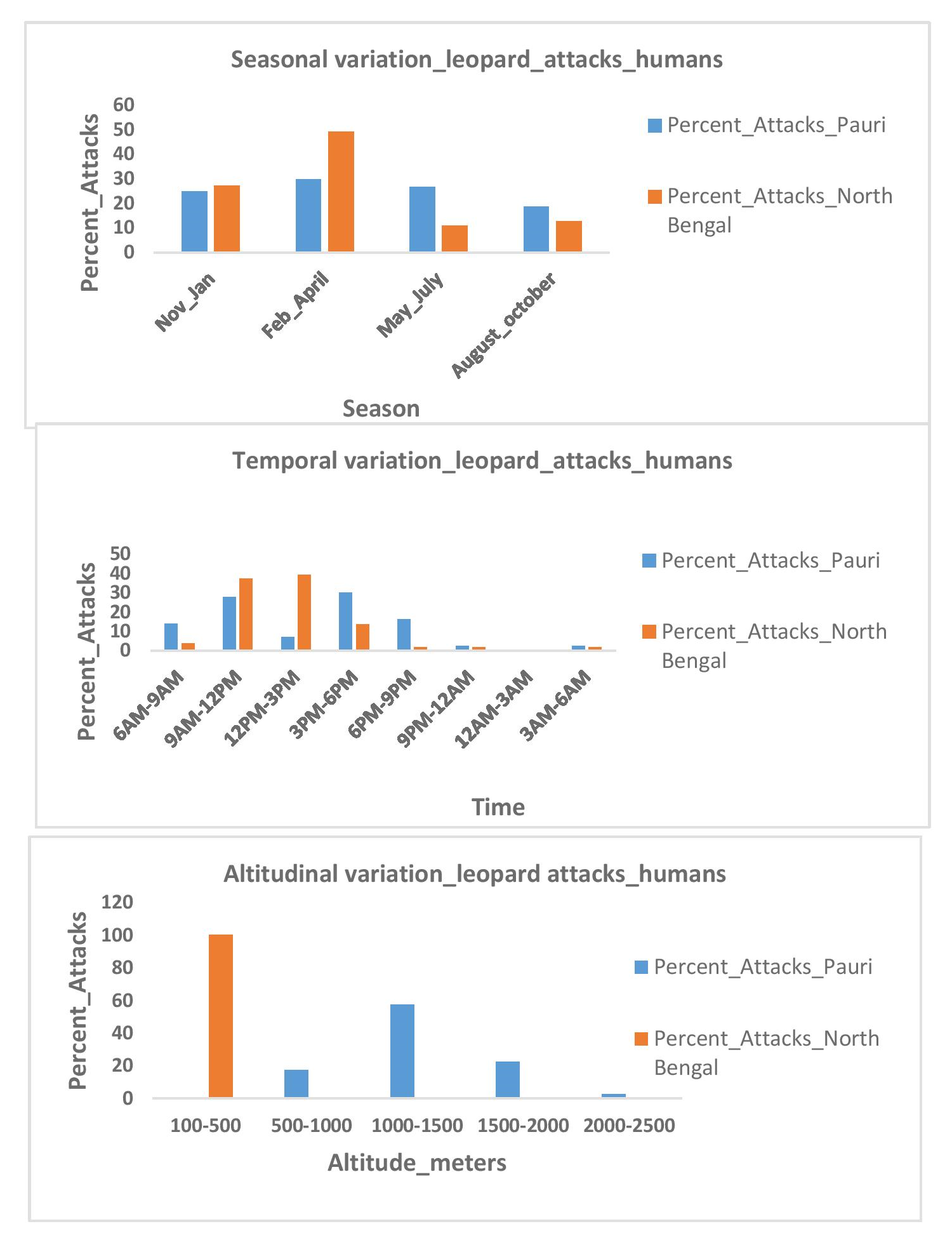

Supplement: S1 Fig — (TIFF) [file pone.0204528.s001.tiff]

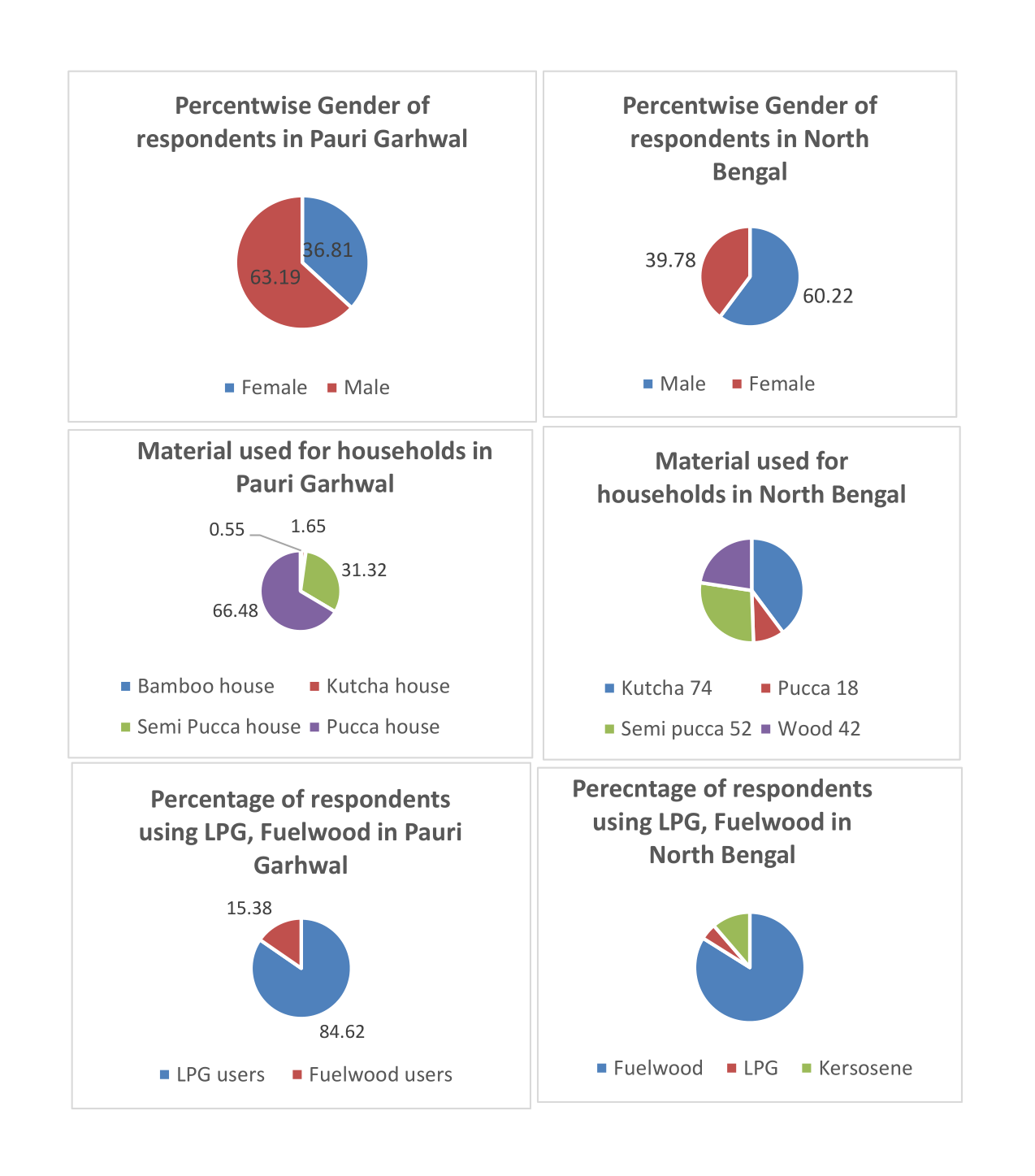

Supplement: S2 Fig — (TIFF) [file pone.0204528.s002.tiff]

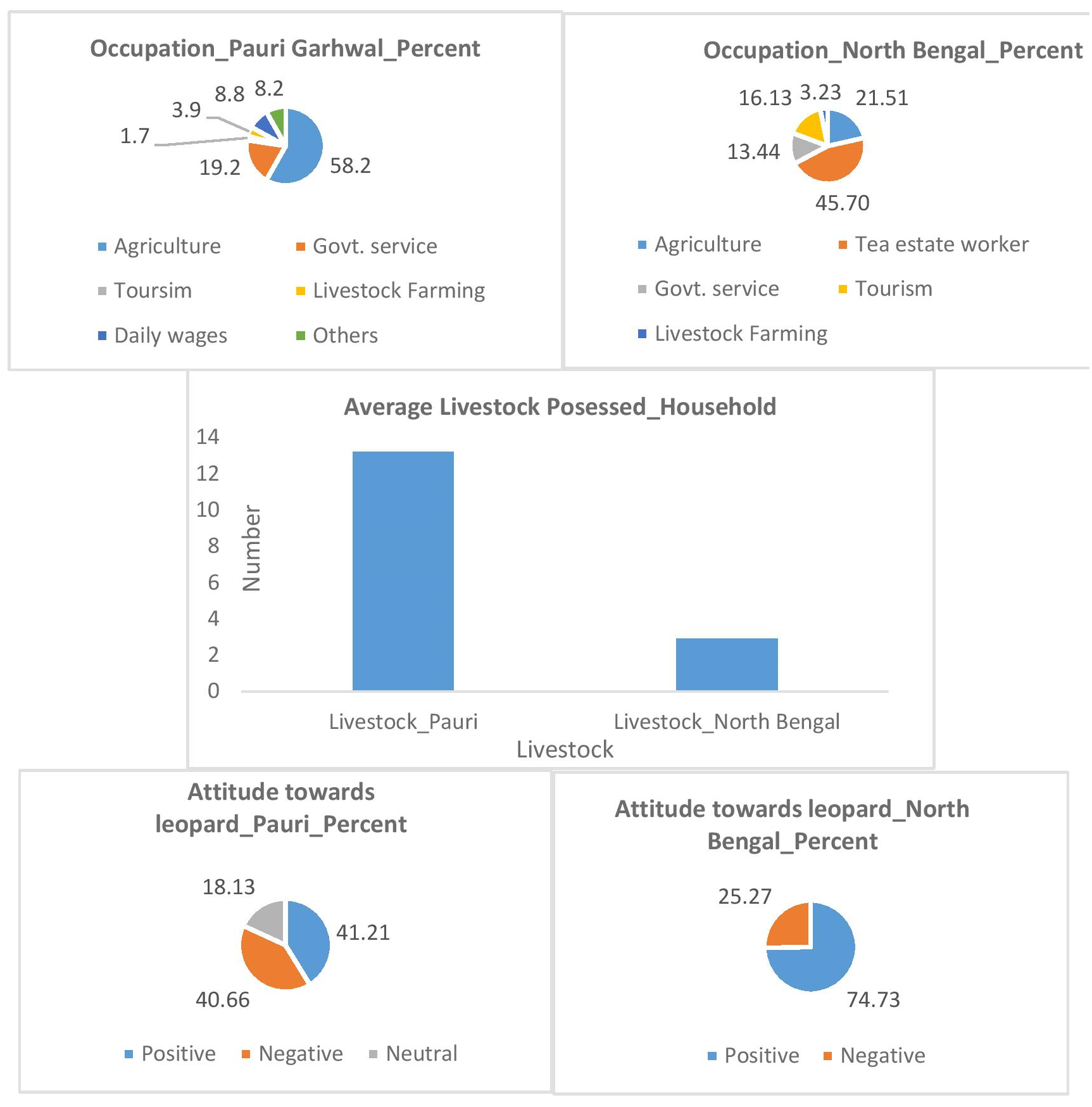

Supplement: S3 Fig — (TIFF) [file pone.0204528.s003.tiff]

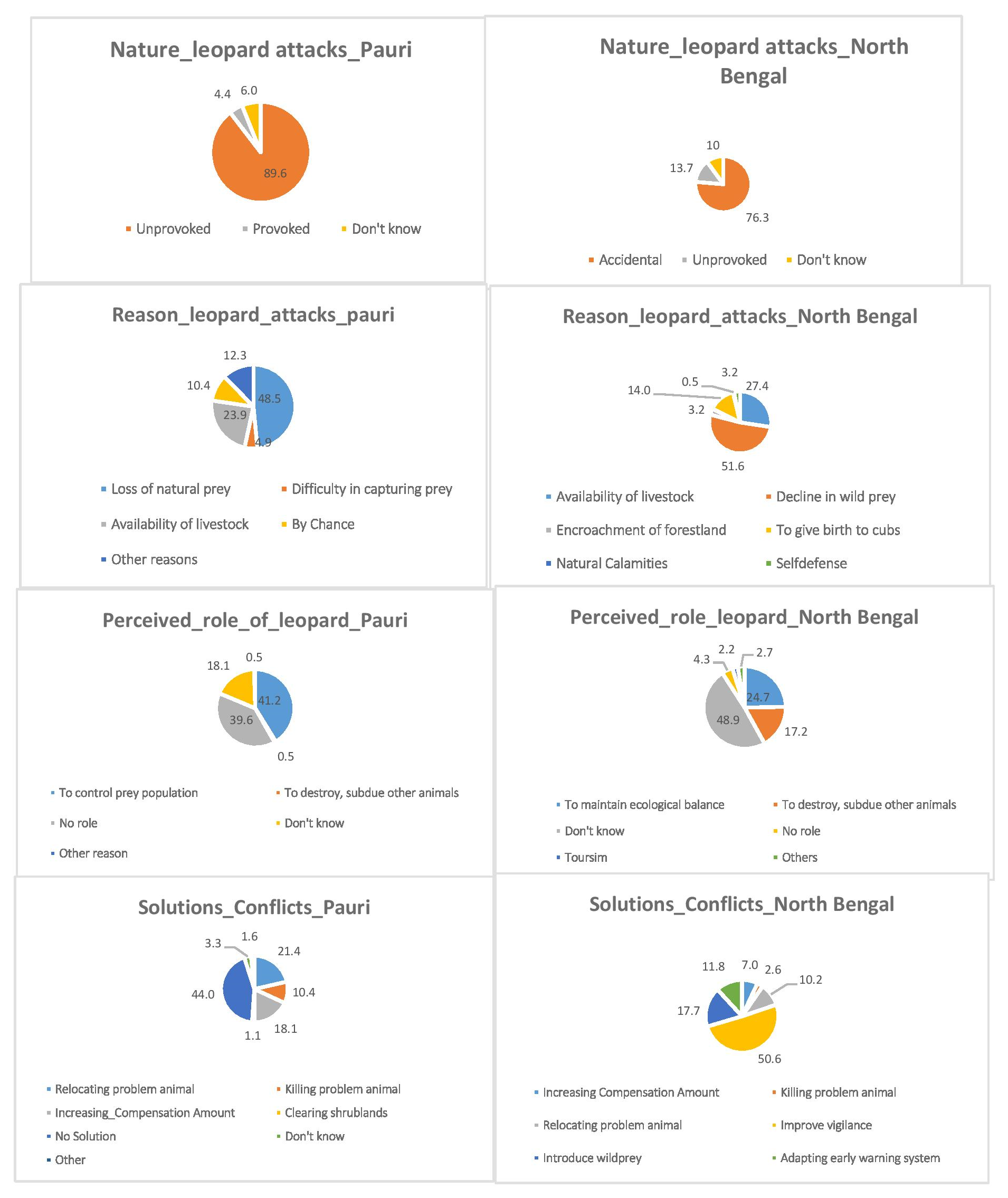

Supplement: S4 Fig — (TIFF) [file pone.0204528.s004.tiff]
