# Supplementary material for: Understanding drivers of human-leopard conflicts in the Indian Himalayan region: Spatio-temporal patterns of conflicts and perception of local communities towards conserving large carnivores
Source: PLoS One. 2018 Oct 5;13(10):e0204528. doi: 10.1371/journal.pone.0204528 (PMC6173383; doi:10.1371/journal.pone.0204528)
Supplement: S1 Appendix — This datasheet was used to record socio-economic and perception data of local communities in Pauri Garhwal and North Bengal. (DOCX) [file pone.0204528.s005.docx]

**QUESTIONNAIRE SURVEY**

Village Name Date:

**SECTION 1:** **Interviewer's assessment (Do a visual assessment of the interviewee)**

GPS Location:

Name of Interviewee………………………………………………………………………..

Note: Do not ask interviewee questions from this section

**1.1.** **Based a visual assessment of the interviewee, answer the following**:

Age ____ (b) gender: ( ) Male ( ) Female

Number of persons in household (gender wise adult and kids) _________________

**1**.**2. What is your educational level?**

( ) Illiterate ( ) Primary school incomplete ( ) Primary school complete ( ) Secondary school incomplete ( ) Secondary school complete ( ) Undergraduate course incomplete ( ) Undergraduate course complete

**1.3.** **Religion & Caste Status:**

**SECTION 2**: **Household characteristics**

**2.1. What type of house do you have?**

(a) Pucca (b) semi-pucca (c) kutcha (d) Bamboo/Wood

**2.2. What type of roof does your house have?**

(a) Thatch (b) Tinned/cement sheets (c) Mud baked tiles (old/new type) (d) Concrete

**2.3. Do you have electricity connection?**

(a) Yes (b) No

**2.4. What source of fuel do you use for cooking purpose?**

(a) LPG gas (b) Kerosene (c) Bio-gas (d) Fuel wood

**2.5. How long have you lived in this place?**

(a) First generation (b) Second generation (c) More than two generations

**2.6. What is/are your source/s of livelihood? (List in terms of priority if more than one)**

(a) Service (government/private) (b) Agriculture (c) Livestock farming (d) Daily wages/Tea estate worker (e) Hotel/Tourism (e) Others

**2.7. What is your average annual income?**

**2.8 Do you have toilets? How far it is from the household?**

**SECTION 3**

**3.1.** Livestock

**Do you own livestock?**

(a) Yes (b) No

Purpose of Keeping Livestock: meat ( ), agriculture ( ) milk ( ) personal consumption ( ) source of income

**3.2. How much livestock do you own? (Mention numbers for each)**

1. Sheep:
2. Buffalo:
3. Goats:
4. Cattle:
5. Poultry:
6. Others (specify):

**3.3. Do you lose livestock to disease or carnivore attack if yes, which carnivore? and how many per year? (Financial and number both)**

**Specify livestock’s & their number/ disease/ carnivore involved/ year**

Livestock:________________No:_____________Disease: ___________________________ Carnivore Involved _________________ Year:______________Economic Loss:_______________________

**SECTION 4: Agriculture**

**4.1. Do you own agricultural land?**

(a) Yes (b) No

If ‘yes’ to the above, is it irrigated? Yes/No___________

**4.2. What is the size of your land holding? (bighas/kathas) __________**

**4.3. How many crops do you get from your land in one year?** (a) 0 (b) 1 (c) 2 (d) 3

**Personal Comments**

**SECTION 5: Perceptions about wildlife**

**5.1. Please, indicate your attitude towards leopard:** {Like/ Indifferent/ Dislike/ Do not know} Leopard ( )

**5.2.** **Do you think conversion of forest area into tea estate was useful for your livelihood?**

If yes then why? If no then why?

**5.3. What is the role of Leopard in the wild?**

1. To destroy/subdue or prey on other animals
2. To maintain ecological balance
3. Do not know

**6.1. Whether leopard attacks are accidental or predatory?**

1. Accidental (Provoked)
2. Predatory(Unprovoked)
3. Don't Know

**6.2. How is it like staying with wildlife? Statement……..**

**6.3. What items do you use as self-defense when you are working in tea estate/agriculture land?**

**6.4 Do you think conversion of forest areas into scrublands has intensified leopard attacks?**

If yes then why? If no then why?

**6.5. Why do conflict happen?**

1. Loss/decline in wild prey
2. Difficult in capturing natural prey
3. Easy food i.e. Livestock
4. Confusion with forest land
5. Encroachment & Fragmentation of forested habitat
6. By chance
7. to give birth to litter

**6.7 Why do you think people have abandoned the hills?**

(a) Lack of sustainable livelihood (b) lack of irrigation water for agriculture (c) increase shrub/ weed cover (d) over abundance of wildlife (e) Harsh terrain and climate (f) Poor health and education facilities

**6.8. Regarding the predation of livestock and humans killed/injured by leopard** **what would you suggest for solving the problem?**

1. Using preventive methods in the property (use predator proof-enclosures during night, use electric fences to surround pastures and/or enclosures, keep herds away from the forest, etc.
2. Killing the problem animal(s)
3. Financial compensation for livestock losses and human casualties by predators.
4. Relocating the problem animal(s).
5. Removing/eradicating all problem animals from protected areas.
6. Restructuring the food chain (reintroduction of wild prey species).
7. Others
8. No solution
9. Do not know
10. Increasing vigilance while working
11. Clearing shrub/weed around villages
12. Putting up streetlights
13. Using early warning systems
